# Predicting of Sentinel Lymph Node Status in Breast Cancer Patients with Clinically Negative Nodes: A Validation Study

**DOI:** 10.3390/cancers13020352

**Published:** 2021-01-19

**Authors:** Annarita Fanizzi, Domenico Pomarico, Angelo Paradiso, Samantha Bove, Sergio Diotaiuti, Vittorio Didonna, Francesco Giotta, Daniele La Forgia, Agnese Latorre, Maria Irene Pastena, Pasquale Tamborra, Alfredo Zito, Vito Lorusso, Raffaella Massafra

**Affiliations:** 1 Struttura Semplice Dipartimentale di Fisica Sanitaria, I.R.C.C.S. Istituto Tumori “Giovanni Paolo II”, Viale Orazio Flacco 65, 70124 Bari, Italy; a.fanizzi@oncologico.bari.it (A.F.); domenico.pomarico@hotmail.it (D.P.); v.didonna@oncologico.bari.it (V.D.); p.tamborra@oncologico.bari.it (P.T.); r.massafra@oncologico.bari.it (R.M.); 2 Oncologia Medica Sperimentale, I.R.C.C.S. Istituto Tumori “Giovanni Paolo II”, Viale Orazio Flacco 65, 70124 Bari, Italy; a.paradiso@oncologico.bari.it; 3 Dipartimento di Matematica, Università degli Studi di Bari, 70121 Bari, Italy; s.bove9@studenti.uniba.it; 4 Struttura Semplice Dipartimentale di Chirurgia, I.R.C.C.S. Istituto Tumori “Giovanni Paolo II”, Viale Orazio Flacco 65, 70124 Bari, Italy; sergiodiotaiuti@gmail.com; 5 Unità Operativa Complessa di Oncologia Medica, I.R.C.C.S. Istituto Tumori “Giovanni Paolo II”, Viale Orazio Flacco 65, 70124 Bari, Italy; f.giotta@oncologico.bari.it (F.G.); a.latorre@oncologico.bari.it (A.L.); vitolorusso@me.com (V.L.); 6 Struttura Semplice Dipartimentale di Radiologia Senologica, I.R.C.C.S. Istituto Tumori “Giovanni Paolo II”, Viale Orazio Flacco 65, 70124 Bari, Italy; 7 Unità Operativa Complessa di Anatomia Patologica, I.R.C.C.S. Istituto Tumori “Giovanni Paolo II”, Viale Orazio Flacco 65, 70124 Bari, Italy; m.pastena@oncologico.bari.it (M.I.P.); a.zito@oncologico.bari.it (A.Z.)

**Keywords:** sentinel lymph node, early breast cancer, decision support system, OSNA, clinically negative lymph node, CancerMath

## Abstract

**Simple Summary:**

Sentinel lymph node biopsy procedure is time consuming and expensive, but it is still the intra-operative exam capable of the best performance. However, sometimes, surgery is achieved without a clear diagnosis, so clinical decision support systems developed with artificial intelligence techniques are essential to assist current diagnostic procedures. In this work, we evaluated the usefulness of a CancerMath tool in the sentinel lymph nodes positivity prediction for clinically negative patients. We tested it on 993 patients referred to our institute characterized by sentinel lymph node status, tumor size, age, histologic type, grading, expression of estrogen receptor, progesterone receptor, HER2, and Ki-67. By training the CancerMath (CM) model on our dataset, we reached a sensitivity value of 72%, whereas the online one was 46%, despite a specificity reduction. It was found the addiction of the prognostic factors Her2 and Ki67 could help improve performances on the classification of particular types of patients.

**Abstract:**

In the absence of lymph node abnormalities detectable on clinical examination or imaging, the guidelines provide for the dissection of the first axillary draining lymph nodes during surgery. It is not always possible to arrive at surgery without diagnostic doubts, and machine learning algorithms can support clinical decisions. The web calculator CancerMath (CM) allows you to estimate the probability of having positive lymph nodes valued on the basis of tumor size, age, histologic type, grading, expression of estrogen receptor, and progesterone receptor. We collected 993 patients referred to our institute with clinically negative results characterized by sentinel lymph node status, prognostic factors defined by CM, and also human epidermal growth factor receptor 2 (HER2) and Ki-67. Area Under the Curve (AUC) values obtained by the online CM application were comparable with those obtained after training its algorithm on our database. Nevertheless, by training the CM model on our dataset and using the same feature, we reached a sensitivity median value of 72%, whereas the online one was equal to 46%, despite a specificity reduction. We found that the addition of the prognostic factors Her2 and Ki67 could help improve performances on the classification of particular types of patients with the aim of reducing as much as possible the false positives that lead to axillary dissection. As showed by our experimental results, it is not particularly suitable for use as a support instrument for the prediction of metastatic lymph nodes on clinically negative patients.

## 1. Introduction

Cancer diagnosis at the early stages is a crucial step for the prevention of metastatic processes when cells spread from the primary tumor by contiguity, via the blood or the lymphatics, settling in other organs or tissues. This event causes an extremely negative impact on both the course of the disease and the long-term survival, the early detection of the disease spread, and the early treatment through medical or surgical therapy can avoid the negative effects of distant dissemination and restore a good prognosis to the patient. This is particularly true in the context of mammary neoplasms for which the early metastasis main route is the axillary lymphatic node.

Lymph node metastatic positivity highlights a non-lethal spread of the disease, generally treated through surgical dissection [1,2,3,4]. A lymph node affected by cancer can present some signs of the disease that can be detected through an ultrasound, Computerized Tomography (CT), or Magnetic Resonance (MR) and, in part, through a clinical exam [5]. Unfortunately, the accuracy of these tests is not absolute and has a large number of false negativity and positivity. In current clinical practice, in the absence of lymph node abnormalities detectable with clinical examination or imaging [1], the guidelines [6] also provide for the removal of the first axillary draining lymph nodes, which are identified by the injection of a radionuclide during surgery for the removal of malignant breast lesions. These lymph nodes are called “sentinel”, identified with a technique known as sentinel node and occult lesion localization (SNOLL), and the subject of a second intra-operative pathological examination known as one-step nucleic acid amplification (OSNA), which currently has sensitivity between 87.5 and 100% and a specificity between 90.5 and 100% [7,8,9,10].

Although the OSNA is time consuming and expensive, it is still the intra-operative exam with the highest performance, but sometimes, surgery is achieved without a clear diagnosis. Moreover, large prospective trials have documented sentinel lymph node bioptic complications including allergic reactions, wound infection, seroma, paresthesias, lymphedema, and hematoma [11,12]. For many patients, sentinel lymph node bioptic is more morbid than partial mastectomy, and therefore, axillary surgery is not considered therapeutic in early stage breast cancer [13,14]. In this context, there is a need to evaluate other less invasive and preferably cheaper diagnostic tools that could replace sentinel lymph node bioptic without compromising patient care diagnosis, so Clinical Decision Support Systems (CDSS) developed with artificial intelligence techniques are essential to assist current diagnostic procedures.

The decision support system can be used joint with surgical activity if a high accuracy in predicting sentinel lymph node status is reached. Specifically, the intervention of biopsy could be avoided and the efficiency of surgical intervention could be reduced in terms of time and costs. Among the different proposals in the literature, CancerMath (CM) [15] is an open source cancer web calculator developed to provide support in the clinical outcome prediction for patients affected by breast cancer, melanoma, and renal cells, as well as accurately evaluate the impact of various treatment choices [3,4]. Among the different implemented calculators, CM also allows calculating the probability of having different positive lymph nodes for breast tumors characterized by a certain dimension estimated according to particular prognostic factors such as age, histological type, grading, and the presence of both estrogen and progesterone receptors.

In this work, with the aim of evaluating the usefulness of this tool in the sentinel lymph nodes positivity prediction for clinically negative patients, we first assessed the CM algorithm classification performance on a sample of patients affected by breast cancer referred to our institute. The innovative approach of our work is to train appropriately the CM algorithm on our kind of patients and adapt it to the task to predict sentinel lymph nodes status in clinically negative patients, therefore tendentially with an early stage tumor. We have evaluated the classification results obtained both by considering the same set of prognostic factors used by the online software and by adding other known prognostic factors such as proliferation marker Ki67 and human epidermal growth factor receptor 2 (Her2).

## 2. Materials and Methods

### 2.1. Experimental Data

**Figure 1:**
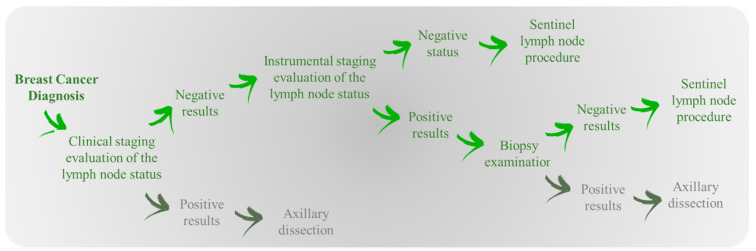
The diagram shows the typical processes of the work-up and interventions used in practice for breast cancer patients with clinically negative lymph nodes.

From 2016 to 2018, we have collected the histological outcome of 993 patients of Istituto Tumori “Giovanni Paolo II” of Bari (Italy) who had negative results after both clinical and instrumental examination and had undergone the OSNA procedure. Specifically, we considered the patients who were clinically negative for lymph nodes and did not have suspicious signs in axillary ultrasound, routine examination in the pre-surgical staging phase of the armpit, or they were negative by fine needle aspiration biopsy following the identification of axillary changes on instrumental examination. In Figure 1, we provided a diagram showing the typical processes of the work-up and interventions used in practice for breast cancer patients with clinically negative lymph nodes.

In our analysis, in addition to the features used by CM software, that is age at diagnosis, histological subtype (ductal, lobular), estrogen receptor expression (ER, Pos/Neg), progesterone receptor expression (PR, Pos/Neg), histological grade (G, Elston-Ellis scale: 1, 2, 3), tumor size (T, measured in mm), and sentinel lymph nodes status (N, Pos/Neg), we used also the cellular marker for proliferation (Ki67, Pos/Neg with cut-off 20%), epidermal growth factor receptor 2 (Her2: negative/positive).

The retrospective observational study was approved by our Institute’s Scientific Board. Before undergoing routine surgery, all patients signed an informed consent form authorizing the use of the removed biological tissue for research purposes according to ethical standards.

### 2.2. Histological Evaluation Procedure

According to the European guidelines in case of uncertain or suspect clinical or radiological exam of the axillary state, the surgeon decides either to operate a lymph nodes axillary dissection during the surgery or, in absence of suspicion, to proceed with the sentinel lymph nodes biopsy evaluation. The OSNA method is an isothermal procedure that employs a nucleic acids rapid amplification technology in order to detect the mRNA expression level in the cytokeratin 19, which is an epithelial cells marker not normally present in lymph node tissue. The OSNA system is in compliance with the European directive about the in vitro diagnostic 98/79/CE (CE-IVD), and it is therefore approved for diagnostic purposes throughout Europe.

The histological examination was performed through multiple sampling with 14–16 G core biopsy under ultrasound guidance. The picked whips were inserted in a container with formalin and sent on the same day to the Pathological Anatomy Department. Histological grade and expression of estrogen receptor (ER), progesterone receptor (PR), human epidermal growth factor receptor 2 (HER2), and Ki-67 antigen associated with cell proliferation were determined by immunohistochemical analysis carried out by the subspecialty department of breast disease in our institute. Specifically, for each sample, the expression of ER, PR, and Ki67 were valued in percentage terms. Tumor grade G was defined by the Elston–Ellis modification of the Scarff–Bloom–Richardson grading system on a three-grade scale. Specifically, based on the duct structures, the size and shape of the nucleus in the tumor cells and mitotic rate were assessed, and a lesion can be of grade G1 (low grade), G2 (intermediate grade) or G3 (high grade), where a lower grade indicates a better prognosis [16].

### 2.3. Algorithm Cancer Math

The CancerMath (CM) algorithm implemented on an online free platform [15] and with an open source code aims at modeling the diffusion of cancer cells belonging to the primary lesion [3,4]. CM estimates the probability of lymph node involvement on the basis of prognostic factors such as cancer mass diameter measurement, age, histological type, grading, and estrogen and progesterone receptors.

Compared to the online CM algorithm [15], the probability of positive sentinel lymph nodes is estimated using the following model:

(1) $$L_{n}\;\approx \;1-\exp(-Q_{n}\;\prod g_{i}{\;D}^{Z})$$

where D is the diameter of the primary tumor mass, g_i_ are the parameters associated with each sub-category of the prognostic factors considered, such as age, grading, histological type, ER (Pos/Neg), PR (Pos/Neg), Q_n_ is an interpolation parameter referred to the whole population, while Z is a parameter set equal to 1 when studying lymph node involvement, as indicated by the authors [3,4]. In practice, the probability estimate is a function of the population diameters whose parameters Q_n_ and g_i_ are previously estimated on a training sample.

Specifically, the value of Q_n_ and the parameters are determined through a training procedure starting from the average probability of spread of a tumor cell toward the sentinel lymph nodes, which is estimated in a sub-sample of W patients corresponding to each of the ranges of the above prognostic factors, and the training procedure is developed in two phases. In the first phase, the Q_n_ value is calculated on the entire sample of the reference population by equating the average probability of spread of a tumor cell with the observed statistical frequency of positive lymph nodes in the entire population, in a numerical way, assuming in this first phase that the parameters g_i_ are all equal to 1.

Subsequently, in the second phase, the newly determined value of Q_n_ is fixed in each sub-population, and one proceeds in the same way for each range of values of the prognostic factors, with the estimation of the probability function of only the diameters of the sub-populations, which produce measures corresponding to the impact of the prognostic factor as a cause statistically independent of the others.

This model is included in a broader framework of predictive tools, and the web-calculator generally returns the probability of lymph node involvement estimated by (1) with respect to the characteristics entered and the estimated parameters of the training dataset. In case of missing data, the parameter corresponding to the feature is equal to 1 so that production is not compromised.

Using the online platform, the parameters are pre-set and estimated on the training population used by the authors, which is heterogeneous by type of patients and characteristics of the mammary tumors considered compared to those of our interest.

Starting from the CM algorithm, we investigated three other classifiers, the first one obtained by retraining the same model on our dataset, obtaining therefore new values for the parameters Q_n_ and g_i_ (A), and the other ones obtained by adding either proliferation marker Ki67 (Pos/Neg) (B), human epidermal growth factor 2 Her2 (Pos/Neg) (C), or both (D).

### 2.4. Performance Evaluation

We have performed the importance feature analysis on 80% of the sample that is on 795 clinically negative patients. Specifically, the training set of hold-out validation sampling was made up of 640 cases with negative sentinel lymph node (control cases) and 155 cases, it was positive at post-operative histological survey. In contrast, the hold-out validation sampling was made up of 145 and 53 cases with negative and positive sentinel lymph node, respectively.

The CM performances computed using the online software, thus using the model parameters g_i_ set by the calculator, were compared with the ones obtained by retraining the classification algorithm CM on our dataset with the same features (classifier A) and then adding separately the two prognostic factors Her2 (classifier B) and Ki67 (classifier C), or both these features (classifier D).

The classification performances of CM on line application and A, B, C, and D models both on hold-out training and the test set are evaluated in terms of Area Under the Curve (AUC) of the Receiver Operating Characteristic (ROC) curve and, once we identified the optimal threshold by Youden’s index on ROC curves [17], we have also calculated:

Accuracy = (TP + TN)/(TP + TN + FP + FN)(2)

Sensitivity = TP/(TP + FN)(3)

Specificity = TN/(TN + FP)(4)

where TP and TN stand for True Positive (number of cases with positive sentinel lymph correctly classified) and True Negative (number of cases with negative sentinel lymph correctly classified), while FP (number of negative cases identified as positive) and FN (number of positive cases identified as negative) are False Positive and False Negative ones, respectively.

Specifically, the prediction performances of A, B, C, and D models obtained on the hold-out training set are evaluated on 100 ten-fold cross-validation rounds and summarized in terms of median, 1st, and 3rd quartile.

## 3. Results

Characteristics of the patients are summarized in Table 1. A total of 993 patients aged between 23 and 92 years (with a median, first, and third quartile of 56, 48, and 66 years, respectively) were included in the study. In total, 208 of these were positive on histological examination of the sentinel lymph nodes, while 785 were negative.

Table 2 shows the performances statistics obtained on 100 ten-fold cross-validation rounds in order to collect statistics on the performances provided on the basis of the patient variation in the training set and in the validation one. The CM calculator reaches a specificity of 73.6% and 75.2% but a sensitivity of 46.4% and 41.5% on the hold-out training and test sets, respectively. By training the same algorithm in cross-validation with the same features on the hold-out training set, there is a countertrend in performances: the sensitivity increases to 72.3% and 73.6%, while the specificity drops to 54.2% and 43.4% in the hold-out training and test sets, respectively.

Since the grading is missing information for some patients of our dataset, as well as the histological type, we trained the model used by CM by including factors related to them—that is Ki67 and Her2, respectively (*p*-values Chi-square test < 0.05) [18,19,20]. However, the inclusion of these features characterizing the tumor can replace any missing information. However, the entering of this information, separately or together, does not significantly improve the general performances.

The score thresholds selected by using Youden’s test on the hold-out training set match for the classifiers A, B, C, and D, and are equal to 0.15, while concerning the online algorithm, the threshold is equal to 0.32. This difference is significant due to the non-representativeness of the sample used to train the online web calculator compared to that of our study. These score thresholds are used to classify the subjects of the hold-out test set.

In order to assess whether the model responds better in particular types of patients than others, in Table 3, we have reported the performances obtained by different trained classifiers performances. We have considered patient sub-samples characterized by either particular prognostic factors or clinical conditions, such as diameter of the primary cancer mass (T1 ≤ 20 mm, 20 mm < T2 ≤ 50 mm), age, grading molecular subtypes by summarizing Er, PR, ki67, and HER2. The performances measured on the result of the CM retrained on our data but with the same features are always greater than the performances of the online CM in terms of sensitivity, except for both patients with tumors having a dimension ranging from 20 to 50 mm (T2) and patients with an age between 45 and 60 years, probably in the menopausal period, for which the online CM reaches a sensitivity of 78% and 77%, respectively.

In order not to burden the discussion, we have not included the performance details for the classifiers obtained by adding Ki67 and Her2, or both. In general terms, the median performances observed in the population sub-samples obtained by the retrained classifiers on our data, both with the same features used by the online CM and by including either Ki67 or Her2, are comparable in terms of sensitivity and specificity, emphasizing once again the absence of a significant difference. However, it should be noted that by retraining the classifier and including the factor Ki67, the sensitivity relative to the tendentially menopausal age patients increases to 83%, while the sensitivity of the more aggressive tumors, with grading 3 and triple negative, increases by including the Her2 factor (94% and 100% respectively), but always at the expense of a loss in specificity.

## 4. Discussion

The use of CDSS for the personalized medicine represents the way to preserve the patients’ safety, the care quality, and its efficiency [21]. Especially in the diagnosis of cancers as complex diseases, the introduction of a multifactorial analysis that is able to exploit both the information associated with the presence of particular biomarkers and the one resulting from the genic expression allows us to obtain optimal chemotherapeutic treatments plans [22].

The tools provided by the use of machine learning are able to deal with the complexity associated with this type of diseases in the mutual optimization of several factors [23,24], which is the reason why different platforms are being developed in order to share not only the simple data but also the same algorithms [25].

In the particular case of pre-operative identification of lymph node metastases in breast tumors for clinically negative patients, the prediction of the lymph node status by means of less invasive procedures than the OSNA procedure is an important task that would also allow us to reduce the relative time and cost of the intervention. For this reason, the development of methods able to detect this condition during the first phases of the cancer mass growth is really important [26].

Concerning the clinical application, the OSNA procedure currently offers a sensitivity range equals to 87.5–100%, while regarding the specificity, the range is equal to 90.5–100% [8,9,10]; therefore, the new algorithms with this purpose have to meet or exceed these thresholds.

The relationship between clinical and genetic data leads to focus also on the biomarkers of the receptors (hormonal) estrogen ER and progesterone PR, the proliferation markers Ki67, and the human epidermal growth factor 2 Her2. Starting with these ones, it is possible to define molecular sub-types that are a fundamental breast cancer characterization based on the genetic expression profile [27,28,29]. Immunohistochemical expression on the basis of the St. Gallen sub-types [28] allowed us to verify a statistically significant association between the clinically negative state and the subtype luminal A, as well as the subtype Her2-positive being associated to a clinically positive state. In our analysis, we focused only on the first of these categories, namely clinically negative patients [2].

The online CM algorithm studied in this work aims to estimate the probability of having positive lymph nodes for breast cancers, although this probability computation is included in a broader framework regarding the oncological patient survival analysis. The algorithm estimates this probability on the basis of prognostic factors such as cancer mass dimension, age, histological type, grading, estrogen and progesterone receptors. The online software has been trained on different tumor kinds characterized by various sizes of the primary lesion, which is a parameter that plays a primary role in the probability computation. For this reason, the online algorithm performance shows disputable results on our dataset composed of clinically negative patients, therefore with low values of the tumor diameter. Even though the new training increases the sensitivity, the algorithm presented in this work still does not reach sufficient values for the case of small diameter tumors. In particular, sub-samples characterized by more aggressive tumors, such as the high tumor grade and triple negative one, or for menopausal age one, the addition of the prognostic factors, Her2 and Ki67, respectively, can help in increasing sensitivity, even though for sparsely populated sub-samples. Anyway, the performances do not allow a clinical application of the algorithm.

It should be emphasized that a limitation of our study is the lack of a large amount of data relating to grading and histology. However, the platform itself provides for the possibility of entering “unknown data” as an answer, and the model is trained to deal with this circumstance. Nonetheless, we also evaluated the insertion of features known to characterize the tumor and related to them without obtaining a significant improvement in performance.

The CM performances have been validated in different studies [30,31,32], but only in [31] did the authors consider the package related to the lymph node involvement probability computation on a sample of the population of the South-Eastern Asia. Specifically, in [31], the authors highlight that the median probability estimated by CM of having a lymph node involvement is equal to 40.6%, underestimating the real one of the samples under study (43.6%).

We want to underline that despite the code being open source, in the works in which the CM algorithm was validated on other populations, the model was not re-trained on the population of interest to estimate new parameters, as we did in this work.

On the basis of the state-of-art, most works propose non-sentinel lymph node status predictive models by using features of a different nature, including nomograms of clinical and pathologic variables [33,34,35,36,37]. On the other hand, the literature is poor in studies aimed at the development of sentinel lymph nodes predictive models [38,39,40]. In [38], the author proposed a prediction model for sentinel lymph node metastasis using genetic features, tumor size, and lymph vascular invasion in ER-positive and HER2-negative (ER+/HER2−) breast cancers, thus reaching an AUC value of 0.883. In [39], the authors developed a prediction model for detecting the negativity of sentinel lymph node in order to reduce additional axillary surgery in patients with ductal carcinoma in situ upstaged to invasive cancer. They reached an AUC value of 75% using histological features, such as multifocality, size, histologic type, grade, lymph vascular invasion, hormone receptor expression, and Ki-67 level.

It should be noted that the estimate of the sentinel lymph nodes positivity probability achieves the best performances when supported by radiomic analysis. The information coming from imaging, such as diffusion wavelet and dynamic contrast-enhanced magnetic resonance imaging (DWI and DCE-MRI), is exploited using logistic regression models [41], convolution neural networks [42], and least absolute shrinkage and selecting operator [43,44]. Nevertheless, clinically negative patients are not generally subjected to magnetic resonance imaging, but only to first-level instrumental investigations such as mammography and ultrasound. Radiomic studies on these imaging categories aimed at the prediction of the lymph node involvement are sparse, unlike the many studies on the detection and characterization of breast lesions [45,46,47,48,49,50,51,52]. In recent studies, both histopathologic and radiomic features are jointly used to predict the probability lymph node metastasis, thus obtaining highly performing results [5,53]. These better performances encourage us to face the lymph node state problem by using both other clinical data types (as multifocality, lymph vascular invasion, and tumor location [54]) and information coming from other sources, such as genetics and above all radiomics.

## 5. Conclusions

In this work, we presented the prediction results of sentinel lymph node positivity obtained using the CancerMath software. As showed by our experimental results, it is not particularly suitable for use as a support instrument for the prediction of metastatic lymph nodes on clinically negative patients, not even re-training it on the sample of our patients’ kind. However, some food for thought emerged for other works in the future: the addiction of the prognostic factors Her2 and Ki67 could help improving performances on the classification of particular types of patients. Furthermore, harmonizing the clinical data with the ones obtained from the other information sources, such as radiomics and genetics [38], will be the aim of our future works [24,44,54]. By achieving high levels of accuracy, the use of such a support system would make it possible to avoid both the sentinel lymph node procedure, by reducing time and costs of surgeries, and also unnecessary axillary dissections for particular types of cancers [40].

## Figures and Tables

**Table 1:** Absolute (percentage) distribution of patients with positive lymph nodes according to considered prognostic factors.

Prognostic Factor	*N*. Patients (%)	*N*. Positive (% of Total)	Prognostic Factor	*N*. Patients (%)	*N*. Positive (% of Total)
Overall	993 (100%)	208 (20.95%)	ER (≥1%)		
Age			negative	121 (12.19%)	16 (13.22%)
21–30	2 (0.20%)	0 (0%)	positive	872 (87.81%)	192 (22.02%)
31–40	61 (6.14%)	14 (22.95%)	PR (≥1%)		
41–50	258 (25.98%)	73 (28.29%)	negative	234 (23.54%)	39 (16.67%)
51–60	292 (29.41%)	62 (21.23%)	positive	759 (80.06%)	169 (22.27%)
61–70	239 (24.07%)	39 (16.32%)	Ki67 (≥20%)		
71–80	126 (12.69%)	18 (14.26%)	negative	664 (66.87%)	132 (19.87%)
81–90	14 (1.41%)	2 (14.28%)	positive	329 (33.13%)	76 (23.10%)
>90	1 (0.10%)	0 (0%)	HER2		
Diameter (mm)			negative	870 (87.61%)	180 (20.69%)
T1 (≤20)	748 (75.33%)	130 (17.38%)	positive	117 (11.78%)	26 (22.22%)
T2 (>20, ≤50)	231 (23.26%)	69 (29.87%)	unknown	6 (0.61%)	2 (33.33%)
T3 (>50)	14 (1.41%)	9 (64.29%)	Grading		
Histologic type			G1	106 (10.68%)	32 (30.19%)
ductal	718 (72.31%)	129 (17.97%)	G2	176 (17.72%)	47 (26.70%)
lobular	64 (6.44%)	17 (26.56%)	G3	115 (11.58%)	30 (26.09%)
unknown	211 (21.25%)	62 (29.38%)	unknown	596 (60.02%)	99 (16.61%)

**Table 2:** Classification performances obtained by online CancerMath (CM) application and by training A, B, C, and D models on our dataset. The prediction performance of A, B, C, and D models obtained on the hold-out training set were evaluated on 100 ten-fold cross-validation rounds and summarized in terms of median, 1st, and 3rd quartile.

Model	Performance Measure	Hold-Out Training Set	Hold-Out Test Set
CM on line	AUC (%)	64.7	68.6
	Acc (%)	68.3	66.2
	Sens (%)	46.4	41.5
	Spec (%)	73.6	75.2
CM features (A)	AUC (%)	68.0 (67.6–68.3)	68.6
	Acc (%)	57.6 (55.4–66.2)	51.5
	Sens (%)	72.3 (58.4–76.7)	73.6
	Spec (%)	54.2 (50.5–67.9)	43.4
CM features + Her2 (B)	AUC (%)	67.6 (67.2–68.0)	67.8
	Acc (%)	56.4 (55.1–62.6)	52.0
	Sens (%)	74.2 (62.9–77.7)	73.6
	Spec (%)	52.1 (50.1–63.7)	42.1
CM features + Ki67 (C)	AUC (%)	67.4 (67.0–67.7)	68.0
	Acc (%)	56.4 (55.2–63.5)	50.5
	Sens (%)	74.2 (61.6–76.5)	69.8
	Spec (%)	52.0 (50.2–64.3)	45.5
CM features + Ki67 + HER2 (D)	AUC (%)	64.1 (63.8–64.6)	65.4
	Acc (%)	58.5 (56.1–61.3)	53.8
	Sens (%)	68.1 (52.5–60.6)	70.4
	Spec (%)	55.9 (63.2–71.9)	48.3

**Table 3:** Median values of classifiers performances evaluated on the hold-out training set in stratification sub-samples.

Characteristic	Sample Size (Pos)	CM on Line		A (CM)	
		Sens	Spec	Sens	Spec
Overall	795 (155)	42%	79%	72%	54%
T1	595 (98)	61%	57%	70%	60%
T2	188 (49)	78%	35%	61%	51%
Age ≤ 45	134 (34)	21%	94%	59%	59%
45 < Age ≤ 60	369 (82)	77%	42%	70%	58%
Age > 60	292 (39)	46%	86%	64%	63%
G1	43 (15)	33%	82%	33%	82%
G2	96 (24)	58%	75%	88%	39%
G3	61 (17)	82%	50%	88%	36%
Luminal A	482 (90)	64%	57%	77%	52%
Luminal B	202 (48)	69%	56%	83%	45%
Her2 pos	37 (6)	50%	74%	50%	77%
Triple negative	68 (9)	89%	31%	100%	20%

## Data Availability

The data presented in this study are available on request from the corresponding author. The data are not publicly available because are propriety of Istituto Tumori ‘Giovanni Paolo II’—Bari, Italy.
